# Color-Stable Blue Light-Emitting Diodes Enabled by Effective Passivation of Mixed Halide Perovskites

**DOI:** 10.1021/acs.jpclett.1c01547

**Published:** 2021-06-24

**Authors:** Hongling Yu, Heyong Wang, Tiankai Zhang, Chang Yi, Guanhaojie Zheng, Chunyang Yin, Max Karlsson, Jiajun Qin, Jianpu Wang, Xiao-Ke Liu, Feng Gao

**Affiliations:** † Department of Physics, Chemistry and Biology (IFM), Linköping University, Linköping 58183, Sweden; ‡ Key Laboratory of Flexible Electronics (KLOFE) & Institute of Advanced Materials (IAM), Jiangsu National Synergetic Innovation Center for Advanced Materials (SICAM), Nanjing Tech University, 30 South Puzhu Road, Nanjing 211816, China

## Abstract

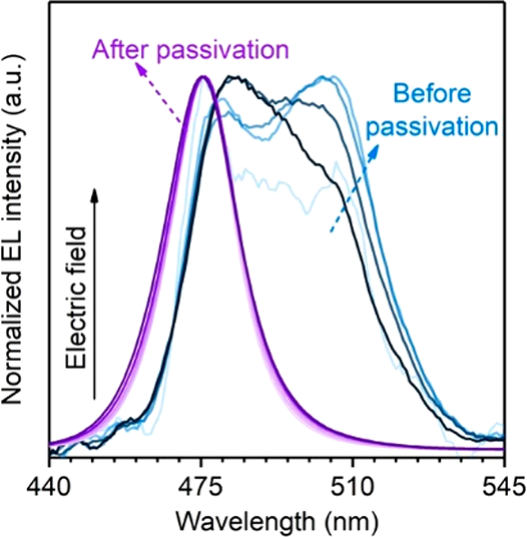

Bandgap tuning through mixing halide anions is one of the most attractive features for metal halide perovskites. However, mixed halide perovskites usually suffer from phase segregation under electrical biases. Herein, we obtain high-performance and color-stable blue perovskite LEDs (PeLEDs) based on mixed bromide/chloride three-dimensional (3D) structures. We demonstrate that the color instability of CsPb(Br_1–*x*_Cl_*x*_)_3_ PeLEDs results from surface defects at perovskite grain boundaries. By effective defect passivation, we achieve color-stable blue electroluminescence from CsPb(Br_1–*x*_Cl_*x*_)_3_ PeLEDs, with maximum external quantum efficiencies of up to 4.5% and high luminance of up to 5351 cd m^–2^ in the sky-blue region (489 nm). Our work provides new insights into the color instability issue of mixed halide perovskites and can spur new development of high-performance and color-stable blue PeLEDs.

Perovskite light-emitting diodes (PeLEDs) have emerged as a promising candidate for solution-processable display and lighting applications because of their exceptional optical and electrical properties, including high photoluminescence quantum yield (PLQY), narrow emission bandwidth, tunable bandgap, and high charge carrier mobility.^1,2^ In the past several years, considerable progress has been made in this field.^3^ State-of-the-art green, red, and near-infrared PeLEDs have achieved high external quantum efficiencies (EQEs) over 20%.^4−7^ In spite of these advances, one of the remaining challenges is to develop blue PeLEDs, which show low performance and hence limit practical applications of PeLEDs.^8−10^

One approach for blue PeLEDs is to develop quantum-confined structures based on pure bromide perovskites; for instance, mixed-dimensional perovskites (mixture of 2D/quasi-2D/3D phases) and perovskite quantum dots (QDs) have been developed.^11−13^ These quantum-confined structures incorporate long-chain organic ligands to suppress the growth of perovskite crystals, keeping at least one direction of the crystals in the range of their Bohr diameters to increase the bandgap.^14−16^ However, it is rather difficult to realize uniform quantum confinement; for instance, multiple phases exist in mixed-dimensional perovskites, sometimes even leading to broad emission and limiting the color purity.^17^ In addition, these quantum-confined structures often suffer from poor charge injection and hence low luminance of the resulting devices because of the insulating long-chain organic ligands.^17−19^

Another approach to develop blue PeLEDs is to employ mixed Br/Cl perovskites. Although continuously tunable emission color with varying halide ions is one of the most appealing features of perovskites,^20^ this mixed halide strategy is often limited by color instability.^21,22^ Most previous reports on mixed Br/Cl-based blue PeLEDs show continuous color shift under operation, usually ascribed to halogen ion migration.^23−26^ Very recently, we and others have demonstrated that homogenizing the halide composition, by either introducing cationic surfactants in the precursor solution^27^ or vapor-assisted crystallization technique during the film crystallization,^10^ can suppress the spectral shift with largely improved device performance.

Herein, we demonstrate that effective passivation of perovskites can also help to stabilize emission color of mixed halide perovskites. We report color-stable blue PeLEDs with additive incorporation into mixed Br/Cl 3D perovskites; this additive is found efficient in passivating the surface defects at grain boundaries, which effectively blocks the ion migration channels to eliminate the color instability of CsPb(Br_1–*x*_Cl_*x*_)_3_ PeLEDs. The optimized device with sky-blue emission peaking at 489 nm delivers a peak EQE of 4.5% and a maximum luminance of 5351 cd m^–2^; these values are 2.9% and 2240 cd m^–2^ for the device with electroluminescence (EL) peaking at 478 nm. As such, our work demonstrates an effective strategy to tackle the color instability issue in mixed halide perovskites and hopefully can spur new development of high-performance and color-stable blue PeLEDs.

The control samples in this study are made from cesium lead mixed Br/Cl perovskites mixed with 4 wt % poly(ethylene oxide) (PEO) (the role of PEO will be discussed later), described as CsPb(Br_1–*x*_Cl_*x*_)_3_ (*x* = 0.38, 0.46, and 0.53). Perovskite thin films are one-step spin-coated from precursor solutions, followed by thermal annealing at 80 °C for 40 min (see Experimental Section in the Supporting Information for details). X-ray diffraction (XRD) measurements are conducted to investigate crystal structures of these films. As shown in Figure S1, the XRD spectra of the perovskite films show diffraction peaks at around 15.4°, 21.9°, and 31.2°, which can be assigned to (100), (110), and (200) planes of CsPb(Br_1–*x*_Cl_*x*_)_3_, respectively.^28^ The intensities of these diffraction peaks are quite low, indicating poor crystallinities of these films.

We characterize basic photophysical properties of these perovskite films with different *x* values. The absorption onset (Figure S2) and bandgap energy *E*_g_ (Figure S3a-c, determined by the Tauc plots^29^) of the CsPb(Br_1–*x*_Cl_*x*_)_3_ films shift to higher energy with increasing Cl content. This observation is consistent with the reported bandgap tuning by substituting halide ions.^30^ Similar to the absorption spectra, the PL spectra (Figure S2) of CsPb(Br_1–*x*_Cl_*x*_)_3_ films with *x* = 0.38, 0.46, and 0.53 show continuous blue-shift with peak wavelength of 482, 471, and 460 nm, respectively.

We fabricate LEDs based on these CsPb(Br_1–*x*_Cl_*x*_)_3_ films by using a device structure of ITO/PEDOT:PSS (40 nm)/perovskite (30 nm)/TPBi (35 nm)/LiF (1 nm)/Al (100 nm) (Figures 1a and S4). The current density–voltage–luminance and EQE characteristics of all the devices are shown in Figure S5a,b, demonstrating low EQEs of less than 0.02%. Figure S5c shows contour maps for the EL spectra for devices with *x* = 0.38, *x* = 0.46, and *x* = 0.53 over the entire working voltage range. We notice that the EL spectra are weak and broad or even have two peaks under different bias voltages (see the normalized EL spectra in Figure 1b–d). The additional emission peak might result from multiple emissive species or ion aggregation following ion migration.^22,31^ The poor spectral stability of these control devices also demonstrates that PEO incorporation hardly improves the color stability. Instead, PEO serves to improve film morphology and facilitate charge injection.^32,33^ We compare the LEDs with and without PEO (Figure S6a–c) and find that PEO incorporation remarkably reduces leakage currents in our LEDs and decreases turn-on voltages (Figure S6a), although it has led to very limited improvement in EQE and EL spectral stability (Figure S6b,c).

**Figure 1:**
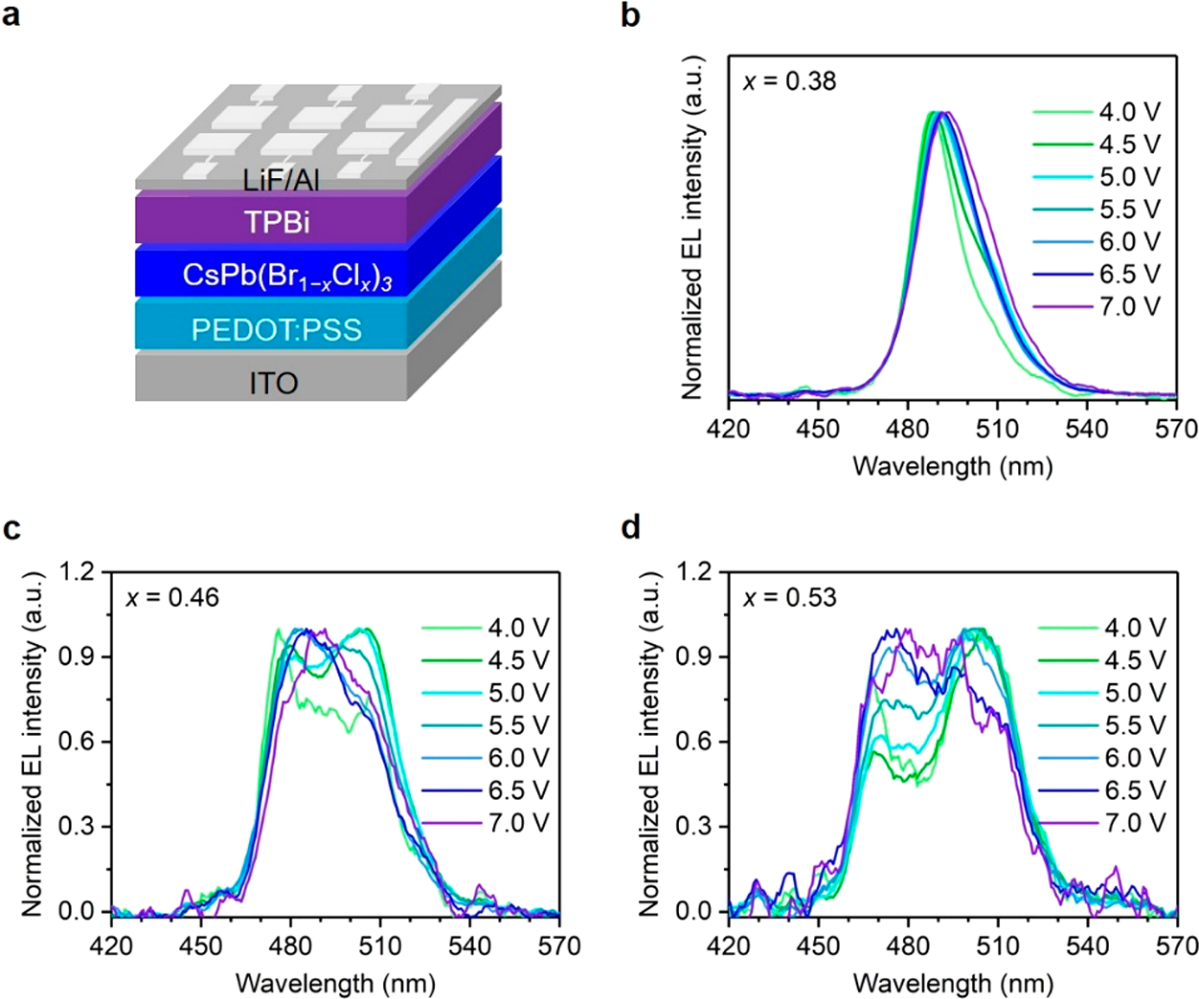
(a) Schematic of the device structure. Normalized EL spectra of devices based on CsPb(Br_1–*x*_Cl_*x*_)_3_ perovskite films with (b) *x* = 0.38, (c) *x* = 0.46, and (d) *x* = 0.53.

With the motivation to obtain spectrally stable emission using mixed Br/Cl 3D perovskite structures, we introduce an additive, organic ammonium chloride salt benzamidine hydrochloride (BHCl; the chemical structure is shown in Figure 2a), into the perovskite precursors to enable the formation of high-quality perovskite crystals with suppressed defect states. The BHCl-treated CsPb(Br_1–*x*_Cl_*x*_)_3_ (*x* = 0.38, 0.46, and 0.53) perovskites are termed *x* = 0.38 (BHCl), *x* = 0.46 (BHCl), and *x* = 0.53 (BHCl) throughout the text, respectively.

**Figure 2:**
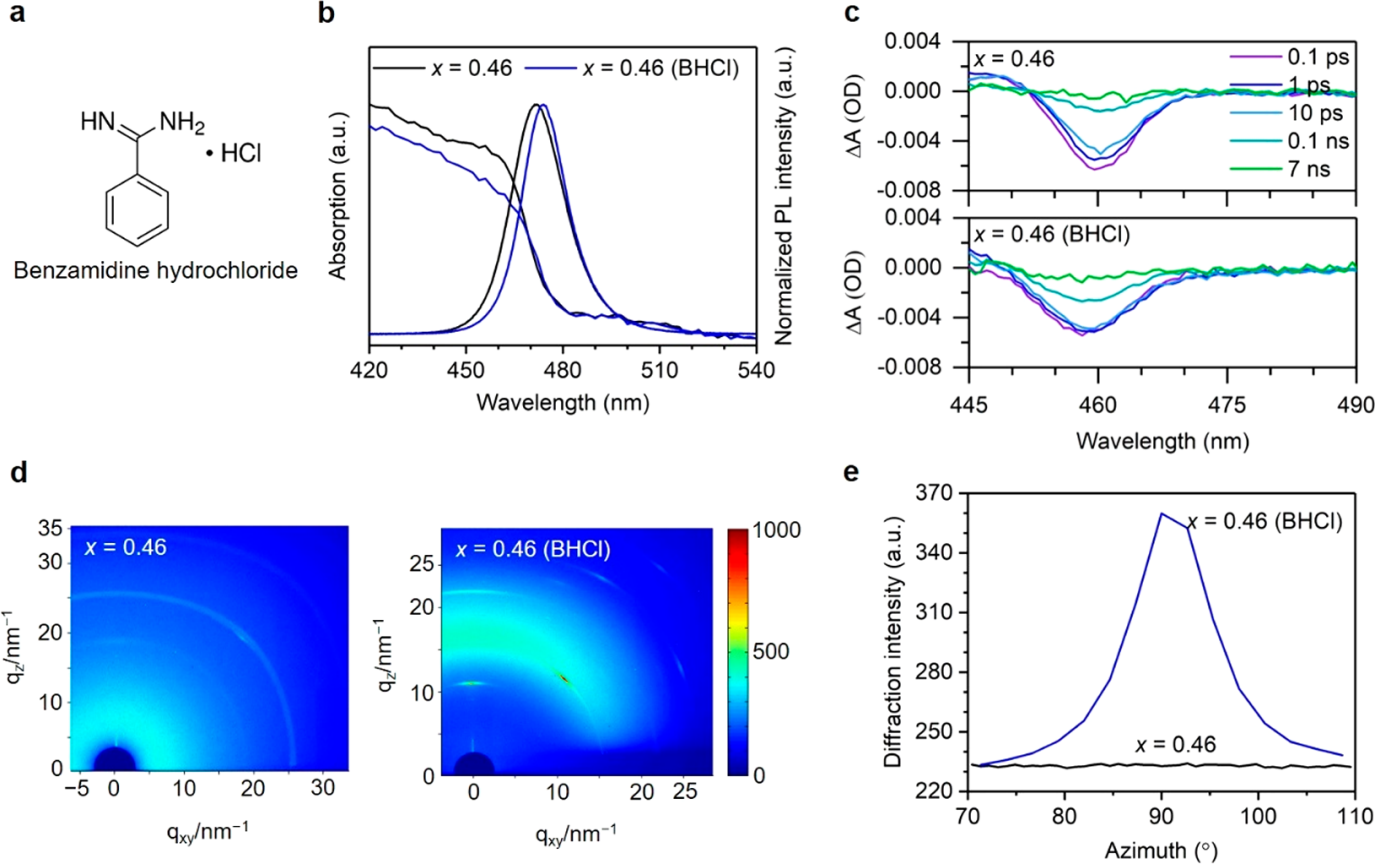
(a) Chemical structure of BHCl. (b) UV–vis absorption and normalized PL spectra (λ_ex_ = 405 nm), (c) TA spectra, (d) GIWAXS patterns, and (e) diffraction intensity of (100) planes versus azimuth angle for control and BHCl-treated CsPb(Br_0.54_Cl_0.46_)_3_ perovskite films.

We perform spectroscopic characterizations to understand whether the introduction of BHCl affects the 3D structures of CsPb(Br_1–*x*_Cl_*x*_)_3_ perovskites. In the case of forming low-dimensional phases after BHCl treatment, we would expect blue shifts in absorption and emission spectra. In contrast, compared to the control films, the BHCl-treated films show almost identical absorption edge, and the PL spectra become narrower with slightly red-shifted peak (Figures 2b and S7a–d), which could be attributed to improved crystalline quality and defect passivation (discussed later).^34^ These absorption and PL spectra suggest that the BHCl-treated films keep the 3D perovskite structures. We provide further evidence based on transient absorption (TA) measurements (Figure 2c). The control and BHCl-treated films show only one single photobleaching peak at 460 and 458 nm, respectively. These results clearly indicate that a single uniform perovskite phase exists in our films and suggest that the additional peaks observed in the pristine films (Figures 1b–d and S5c) result from ion migration and aggregation during the EL measurements.^22^ Ion migration is usually associated with defects in perovskites, especially on the crystal boundaries.^35^

An obvious impact of the BHCl on the perovskite, as we can determine from structural characterisations, is that BHCl helps to significantly enhance crystallization of perovskites. XRD results (Figure S8) show that the intensities of the dominant peaks have largely increased by incorporating BHCl, suggesting an enhancement of the crystallinity or formation of textures in the BHCl-treated films compared to the control films (Figure S1). In addition, the XRD results (Figure S8) show diffraction peaks only from 3D structures, further confirming the absence of low-dimensional phases.^36^

We further conduct grazing incidence wide angle X-ray scattering (GIWAXS) measurements to understand the effect of BHCl on the perovskite crystallization. As shown in Figure 2d, the diffraction pattern in the control film is nearly diffraction rings, and the intensity along the azimuth angle of each diffraction rings shows only slight fluctuations. After BHCl incorporation, an obvious enhancement of diffraction intensity occurs as indicated by the bright yellow/red spots; the diffraction signals located at *q* = 10.7, 15.5, and 21.3 nm^–1^ are assigned to (100), (110), and (200) crystal planes.^10^ In addition, the diffraction intensity at different azimuth angles presents a preferred orientation along the vertical direction. To analyze the evolution of microstructural arrangement, we study the variation of diffraction intensity of the perovskite (100) plane along the vertical direction. As depicted in Figure 2e, the diffraction intensity at the azimuth angle of around 90° increases obviously in the BHCl-treated film. Considering the geometric structure of (100), we ensure that BHCl promotes microstructural arrangements along the out-of-plane direction which finally results in high crystallinity with long-range order. The scanning electron microscopy (SEM) results (Figure S9a–f) also show that the BHCl-treated perovskite grains grow differently from those of the control films.

In addition to enhanced crystallinity, the incorporation of BHCl helps to reduce defect densities, as evidenced by time-resolved PL and power-dependent PLQY measurements. Compared with the control film, the BHCl-treated sample shows longer PL lifetime (Figure 3a); in addition, the BHCl-treated sample shows enhanced PLQYs across a wide range of power density, with a peak PLQY of 10.4% (Figure 3b). These results indicate that BHCl incorporation leads to reduced defect densities, which can result from enhanced crystallization and effective defect passivation. Because the incorporation of BHCl maintains the 3D structures of CsPb(Br_1–*x*_Cl_*x*_)_3_, the passivation is supposed to be at the crystal boundaries of these perovskites.^37^

**Figure 3:**
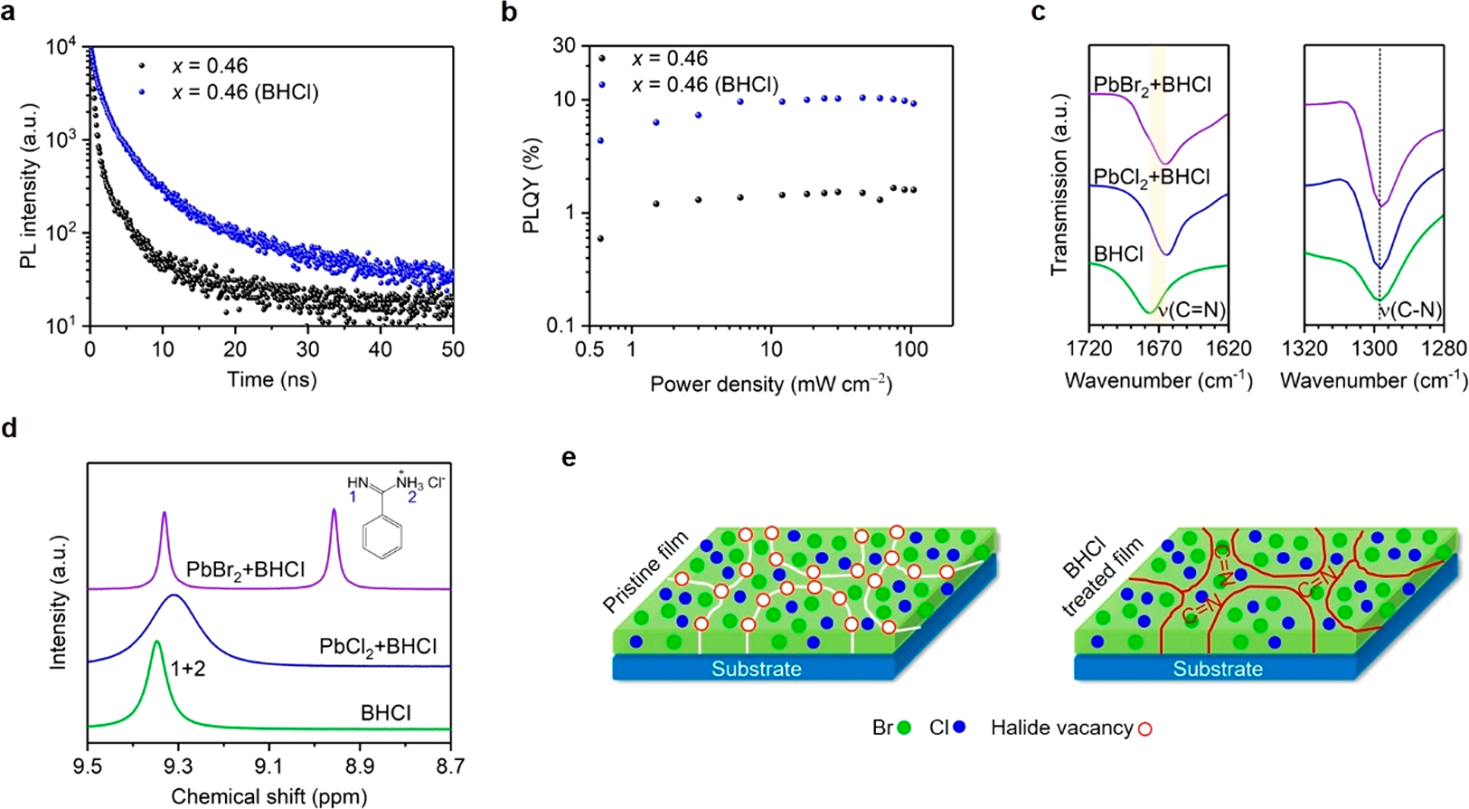
(a) PL decay curves and (b) power-dependent PLQYs of control and BHCl-treated CsPb(Br_0.54_Cl_0.46_)_3_ perovskite films. (c) FTIR spectra of pure BHCl, PbCl_2_ + BHCl, and PbBr_2_ + BHCl films. (d) ^1^H NMR spectra of pure BHCl, PbCl_2_ + BHCl, and PbBr_2_ + BHCl mixed solutions. (e) Schematic illustration of BHCl incorporation induced surface passivation.

In order to understand how BHCl passivates the defects, we perform Fourier-transform infrared (FTIR) measurements (Figure 3c). The C=N stretching (ν(C=N), 1676 cm^–1^)^38,39^ of the BHCl unit shifts to lower wavenumbers in the PbCl_2_ + BHCl and PbBr_2_ + BHCl mixed films. However, C–N stretching (ν(C–N), 1298 cm^–1^)^40^ remains unchanged with respect to that of pure BHCl. These results indicate that the C=N group in BHCl can interact with uncoordinated Pb atoms of PbX_2_. This interaction is further confirmed by ^1^H nuclear magnetic resonance (^1^H NMR) measurements (Figure 3d). Compared to pure BHCl solution, the resonance signal of δ = 9.35 ppm (H^(1+2)^) undergoes a significant broadening or even splitting in PbCl_2_ + BHCl and PbBr_2_ + BHCl mixed solutions, respectively. Hence, the interaction between BHCl and uncoordinated Pb contributes to surface passivation of the perovskites.^41,42^

We propose a model to interpret the differences in crystal grains of control and BHCl-treated films (Figure 3e). In spin-coated CsPb(Br_1–*x*_Cl_*x*_)_3_ perovskite films, a large number of surface defects, including halide vacancies, exist at grain boundaries, which could provide pathways for halide ion migration and result in low-energy emissions.^35,43,44^ After BHCl incorporation, the perovskite crystals are preferentially oriented with reduced defect density. More importantly, BHCl could create surface-passivated grains, suppressing the ion migration channels.

Encouraged by efficient passivation and enhanced crystallization enabled by BHCl incorporation, we are motivated to move forward to fabricate color-stable blue PeLEDs based on BHCl-treated CsPb(Br_1–*x*_Cl_*x*_)_3_. The contour maps in Figure 4a and normalized EL spectra in Figure S10a–c clearly indicate excellent EL spectral stability of the devices. As further indicated in Table S1, the devices show almost constant Commission Internationale de l’Eclairage (CIE) coordinates with increasing voltage. In addition, BHCl incorporation, particularly for the films with *x* = 0.38 and 0.46, suppresses LED leakage currents and results in higher luminance and efficiency (Figures 4b,c and S5a,b). Thus, in addition to enhanced crystallinity and defect passivation, BHCl incorporation may also act as a thin insulating layer together with PEO, suppressing electrical shunts that could have occurred in LEDs with discontinuous perovskite films.^6^ As such, the control device with *x* = 0.38 demonstrates low EQE of less than 0.02% and luminance of less than 60 cd m^–2^; with BHCl incorporation, the device with *x* = 0.38 (BHCl) exhibits a peak EQE of 4.5% and high luminance of 5351 cd m^–2^. Statistical histograms of maximum luminance and EQE of the devices collected from 48 devices (Figure 4d,e) illustrate good reproducibility of our devices.

**Figure 4:**
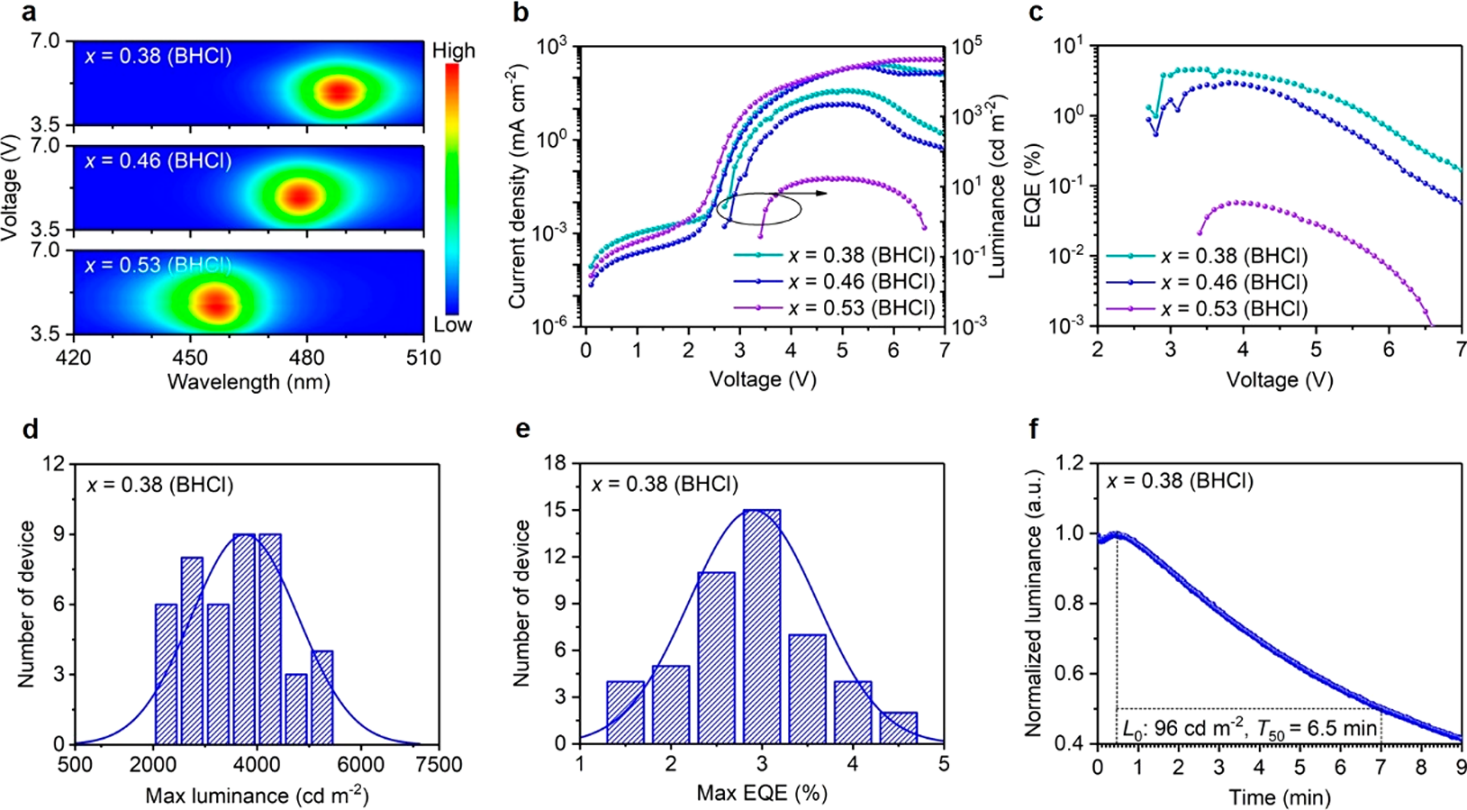
(a) Contour plots of voltage-dependent EL spectra across the entire working range and (b) current density–voltage–luminance and (c) EQE–voltage curves of devices based on BHCl-treated CsPb(Br_1–*x*_Cl_*x*_)_3_ perovskite films. Histograms of maximum (d) luminance and (e) EQE for *x* = 0.38 (BHCl)-based devices. (f) Operating lifetime of *x* = 0.38 (BHCl)-based device under a constant current density of 5 mA cm^–2^.

Figure 4f shows the operational stability of an *x* = 0.38 (BHCl)-based LED, measured at a constant current density of 5 mA cm^–2^ with an initial luminance of 96 cd m^–2^. The luminance value decays to 50% of its initial value (*T*_50_) after 6.5 min. Most importantly, the device shows excellent color stability under operation; the peak wavelengths of EL spectra remain identical during the device stability test (Figure S11). The BHCl-treated films also exhibit excellent spectral stability upon continuous light illumination; after 1 h illumination of 405 nm laser light, no PL shift is observed in these films except for a decrease of the PL intensity (Figure S12a-c).

In conclusion, we have found that the color instability issues in CsPb(Br_1–*x*_Cl_*x*_)_3_ 3D structures appear in the form of additional low-energy emissions, which are related to surface defects at grain boundaries. We prove that such detrimental emissions can be effectively eliminated by passivation through incorporating passivating agents into the precursors. By incorporating BHCl as an additive that can modulate the crystallization process of the perovskite grains and generate surface-passivated grain boundaries, we present color-stable CsPb(Br_1–*x*_Cl_*x*_)_3_ PeLEDs with maximum EQEs of up to 4.5% and high luminance of up to 5351 cd m^–2^ in the sky-blue region (489 nm). Our work provides new insights into the color stability of mixed halide perovskites and sheds light on the development of high-performance and color-stable blue PeLEDs based on CsPb(Br_1–*x*_Cl_*x*_)_3_ 3D structures.

## Supporting Information Available

Detailed experimental methods, XRD patterns, absorption spectra, PL spectra, SEM images, and EL results (PDF)
