# Supplementary material for: Color-Stable Blue Light-Emitting Diodes Enabled by Effective Passivation of Mixed Halide Perovskites
Source: J Phys Chem Lett. 2021 Jun 24;12(26):6041–7. doi: 10.1021/acs.jpclett.1c01547 (PMC8273884; doi:10.1021/acs.jpclett.1c01547)
Supplement: Supplementary file 1 — jz1c01547_si_001.pdf [file jz1c01547_si_001.pdf]

Supporting information

Color-Stable Blue Light-Emitting Diodes Enabled by Effective Passivation of Mixed Halide Perovskites

Hongling Yu[1], Heyong Wang[1], Tiankai Zhang[1], Chang Yi[2], Guanhaojie Zheng[1], Chunyang Yin[1], Max Karlsson[1], Jiajun Qin[1], Jianpu Wang[2], Xiao-Ke Liu[1]* and Feng Gao[1]*

[1]*Department of Physics, Chemistry and Biology (IFM), Linköping University, Linköping 58183, Sweden*

[2]*Key Laboratory of Flexible Electronics (KLOFE) & Institute of Advanced Materials (IAM), Jiangsu National Synergetic Innovation Center for Advanced Materials (SICAM), Nanjing Tech University, 30 South Puzhu Road, Nanjing 211816, China*

**E-mail: xiaoke.liu@liu.se; feng.gao@liu.se*

Experimental section

Chemicals

Poly (3, 4-ethylenedioxythiophene) and polystyrene sulfonate acid (PEDOT:PSS) (AL4083) was purchased from Ossila. 1, 3, 5-tris (2-N-phenylbenzimidazolyl) benzene (TPBi) was purchased from Lumtec. LiF and lead bromide ($PbBr_2$) (99.998%) were purchased from Alfa Aesar. Cesium bromide (CsBr), lead chloride ($PbCl_2$), benzamidine hydrochloride (BHCl) 99%, anhydrous dimethyl sulfoxide (DMSO), and poly(ethylene oxide) (PEO) (average M_v 400,000, powder) were purchased from Sigma-Aldrich.

Precursor solution preparation

CsBr, $PbBr_2$, and $PbCl_2$ were separately dissolved in DMSO to get 0.2 M solutions (1.0 M for BHCl). To prepare $CsPb(Br_{1-x}Cl_x)_3$ (where x = 0.38, 0.46, and 0.53, respectively) precursor solutions, the above-mentioned CsBr, $PbBr_2$, and $PbCl_2$ precursor solutions and 4 wt% PEO (over the total amounts of CsBr, $PbBr_2$, and $PbCl_2$) were mixed in the required molar ratios to obtain 0.075 M (Pb^{2+}) solutions. For example, the precursor solution of x = 0.46 was obtained by mixing CsBr, $PbBr_2$, and $PbCl_2$ solutions in a molar ratio of 1.25 : 0.26 : 0.74 with the addition of 4 wt% PEO. The BHCl-treated perovskite precursor solutions were obtained by mixing the corresponding pristine solutions with Pb : BHCl in a molar ratio of 1.0 : 0.5; the $PbBr_2$ and $PbCl_2$ content were adjusted to maintain the x values. For example, the precursor solution of x = 0.46 (BHCl) was obtained by mixing CsBr, $PbBr_2$, $PbCl_2$, and BHCl solutions in a molar ratio of 1.25 : 0.4 : 0.6 : 0.5 with the addition of 4 wt% PEO.

Film preparation

Glass substrates were cleaned using the ultrasonic bath in deionized water for 15 min and then dried by high-speed nitrogen flow, followed by ultraviolet-ozone treatment for 10 min. Then the cleaned substrates were transferred into the N_2-filled glovebox. The perovskite precursor

solutions were deposited onto bare glasses at 3000 rpm for 60 s and then annealed at 80 ^{o}C for 40 min for film characterizations.

Film characterization

Steady-state absorption spectra were measured with a PerkinElmer model Lambda 900. Transient absorption (TA) experiments were conducted using a pump-probe configuration. Laser pulses (800 nm, 80 fs pulse length, and 1 kHz repetition rate) were generated by a regenerative amplifier (Spitfire XP Pro) via a seed laser (femtosecond oscillator, Mai Tai, Spectra Physics). The samples were pumped at a wavelength of 400 nm generated by a BBO crystal as a second harmonic of the laser. For the probe, the super-continuum generation from a thin CaF_2 plate was used. The mutual polarization between the pump and probe beams was set to the magic angle (54.7°) by placing a Berek compensator in the pump beam. During the experiments, gates on the pump intensity and probe spectra were set to avoid the influence of laser fluctuation on the measurements.

Steady-state photoluminescence (PL) spectra were recorded with a 405 nm continuous wave (CW) laser and an Andor spectrometer (Shamrock sr-303i-B, coupled to a Newton EMCCD detector). The power-dependent PL quantum yield (PLQY) was characterized by a typical three-step technique with a 405 nm CW laser and spectrometer in an integrating sphere. The time-resolved PL measurements were performed on an Edinburgh Instruments spectrometer (FLS1000) with a 405 nm pulsed laser.

X-ray diffraction (XRD) was conducted using a Panalytical Empyrean system with Cu Kα source (λ = 1.54 Å). The Grazing Incidence Wide Angle X-ray Scattering (GIWAXS) measurements were performed at BL14B1 beamline, Shanghai Synchrotron Radiation Facility (SSRF). The wavelength of the X-ray was 1.24 Å (10 keV) and an incidence angle of 0.2° was adopted together with an exposure time of 15 s. The diffraction patterns were collected using a

two-dimensional Mar225 detector. The distance between sample stage and detector is about 313 mm, and all the samples were measured under nitrogen atmosphere.

Scanning electron microscopy (SEM, Philips XL30 FEG SEM) was operated at 3 keV to characterize the morphology of the samples. Fourier-transform infrared (FTIR) studies were carried out on a Bruker Vertex 70 spectrometer. The IR spectra were acquired at 4 cm^{-1} resolution. ^{1}H NMR spectra were measured with a JOEL NMR spectrometer (JNM-ECZ400S, 400 MHz, Japan) with DMSO-d_6 as the NMR solvent.

Device fabrication and characterization

ITO-coated substrates were cleaned using the ultrasonic bath in deionized water for 15 min and then dried by high-speed nitrogen flow, followed by ultraviolet-ozone treatment for 10 min. The filtered PEDOT:PSS solution was spin-coated onto the cleaned ITO substrates at 4000 rpm for 30 s and baked at 140 ^{o}C in the air for 15 min. Then the substrates were transferred into N_2-filled glovebox. The perovskite layers were prepared using the same procedure as the film fabrications. Then the films were moved into a vacuum chamber, in which TPBi (35 nm), LiF (1 nm), and Al (100 nm) were deposited at the rates of 1.0 Å/s, 0.2 Å/s, and 3.0 Å/s, respectively, under vacuum of 2×10^{-6} Torr. Characterization of the devices was carried out at room temperature in a nitrogen-filled glovebox. Current density-voltage (J-V) characteristics were recorded by Keithley 2400 source meter, sweeping from zero bias to forward bias with a step of 0.1 V (lasting for 0.8 s at each step for stabilization). Forward-viewing spectral radiant flux was measured by an integrating sphere coupled with a QE65 Pro spectrometer. The active device area is 0.0725 cm^2.

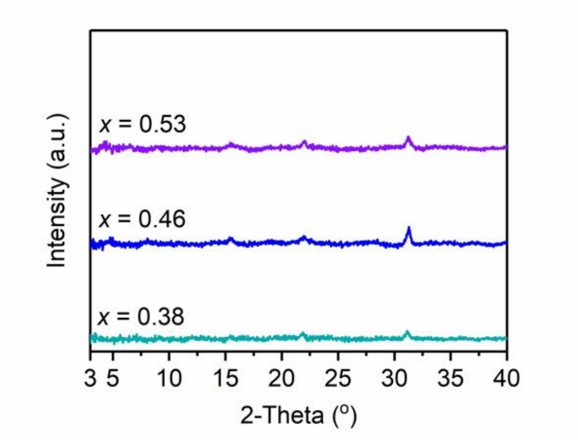

Figure S1. XRD patterns of $CsPb(Br_{1-x}Cl_x)_3$ perovskites with varying x.

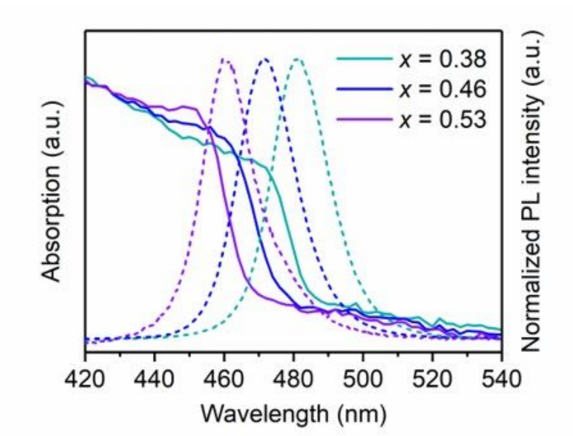

Figure S2. UV-Vis absorption and normalized PL spectra (λ_{ex} = 405 nm) of $CsPb(Br_{1-x}Cl_x)_3$ perovskite films.

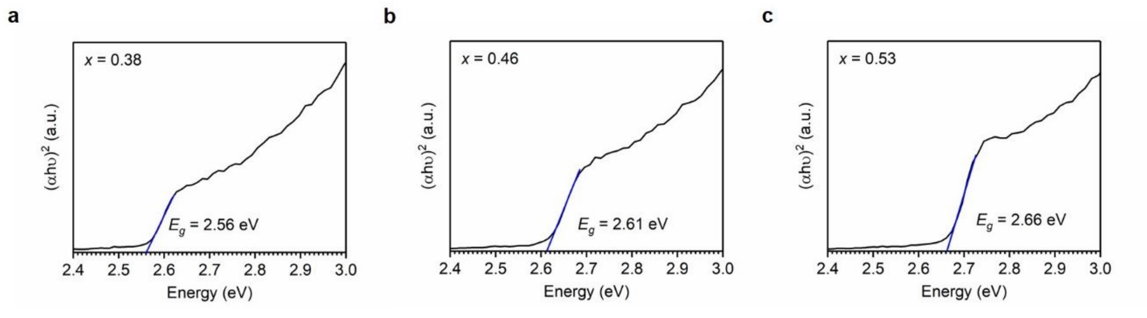

Figure S3. Tauc plots of $CsPb(Br_{1-x}Cl_x)_3$ perovskites with (a) $x = 0.38$, (b) $x = 0.46$, and (c) $x = 0.53$ assuming direct bandgap and showing estimated optical bandgaps from the intercept.

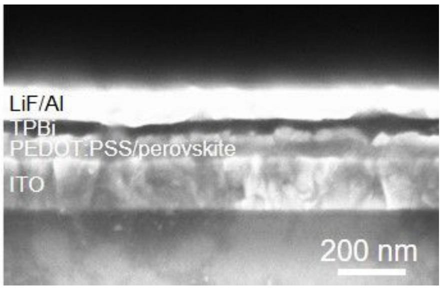

Figure S4. Cross-sectional SEM image of the $CsPb(Br_{1-x}Cl_x)_3$ perovskite film based device.

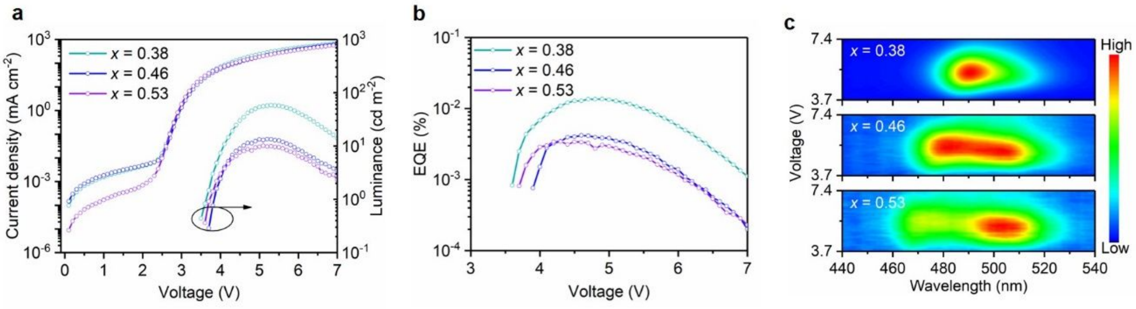

Figure S5. (a) Current density-voltage-luminance curves, (b) EQE-voltage curves, and (c) contour plots of the voltage dependent EL spectra across the entire working voltage range of devices based on $x = 0.38$, $x = 0.46$, and $x = 0.53$ films.

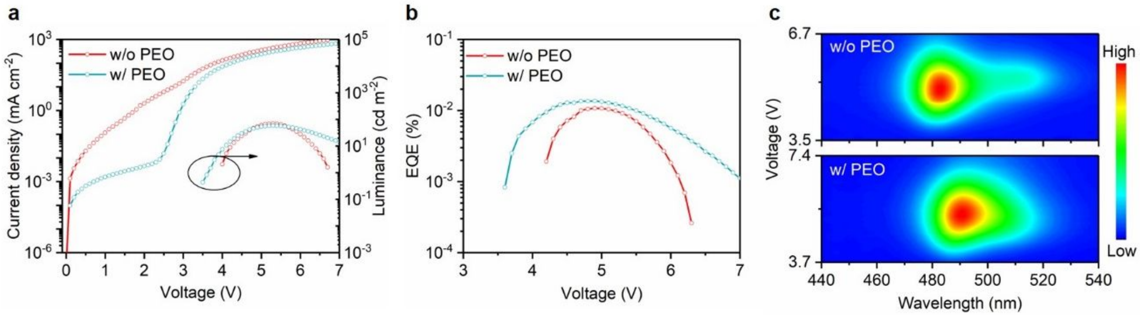

Figure S6. (a) Current density-voltage-luminance curves, (b) EQE-voltage curves, and (c) contour plots of the voltage dependent EL spectra across the entire working voltage range of devices based on $x = 0.38$ films without and with 4 wt% PEO.

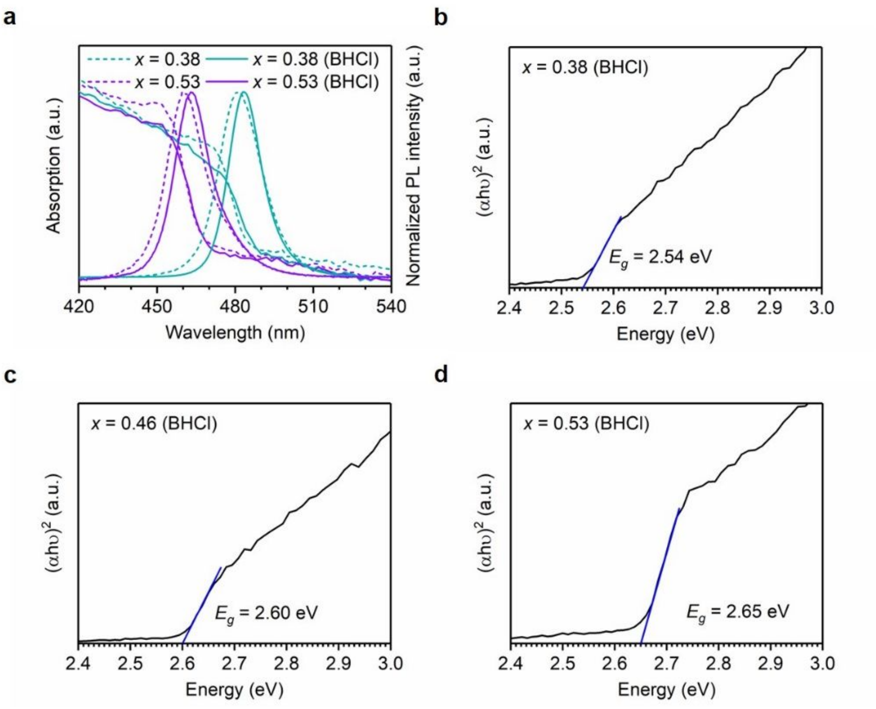

Figure S7. (a) UV-Vis absorption and normalized PL spectra (λ_{ex} = 405 nm) for control and BHCl-treated $CsPb(Br_{1-x}Cl_x)_3$ perovskite films. Tauc plots for (b) x = 0.38 (BHCl), (c) x = 0.46 (BHCl), and (d) x = 0.53 (BHCl) perovskite films.

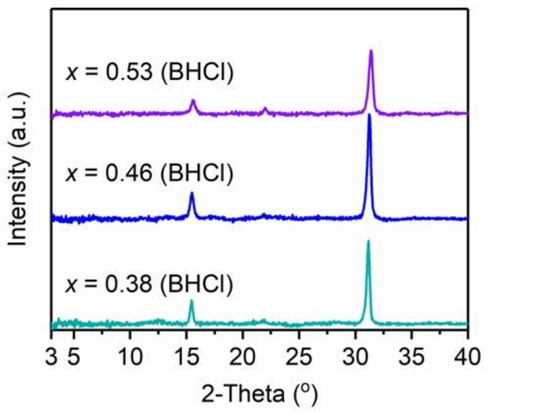

Figure S8. XRD patterns of BHCl-treated $CsPb(Br_{1-x}Cl_x)_3$ perovskite films.

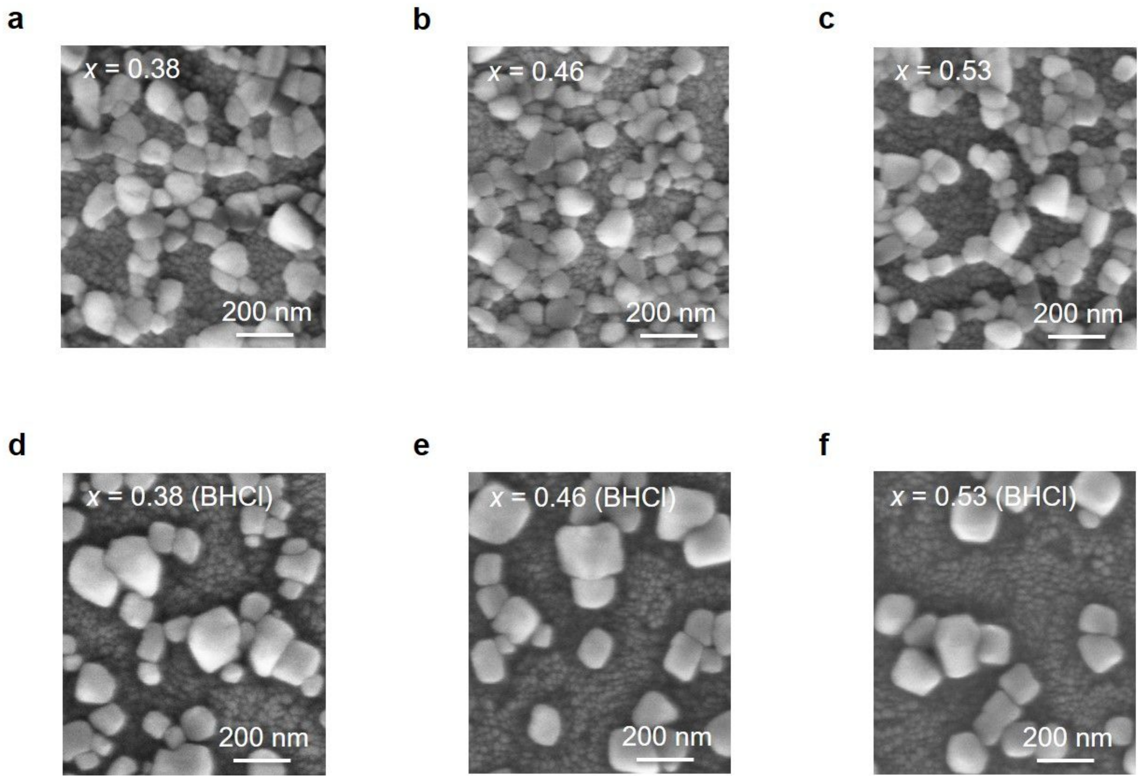

Figure S9. SEM images of control and BHCl-treated $CsPb(Br_{1-x}Cl_x)_3$ perovskite films.

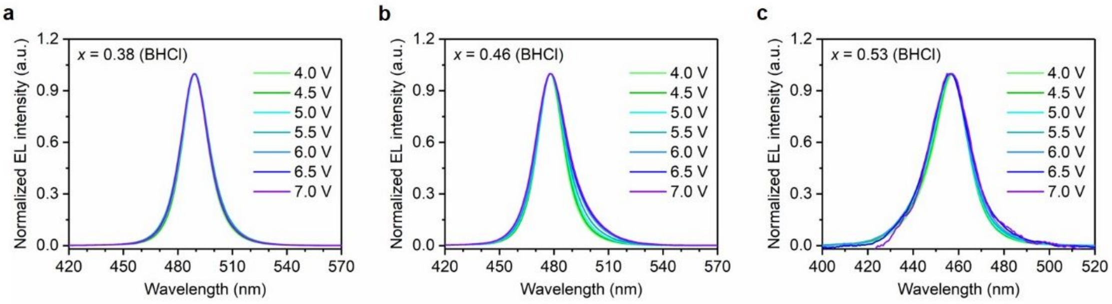

Figure S10. Normalized EL spectra of devices based on (a) x = 0.38 (BHCl), (b) x = 0.46 (BHCl), and (c) x = 0.53 (BHCl) films.

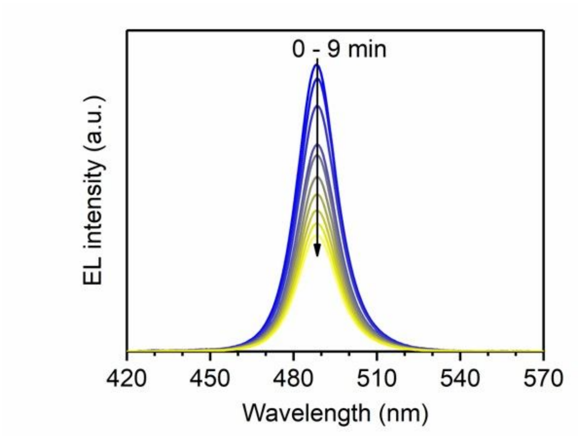

Figure S11. EL spectra of $x = 0.38$ (BHCl)-based device under a constant current density of 5 mA cm^{-2}.

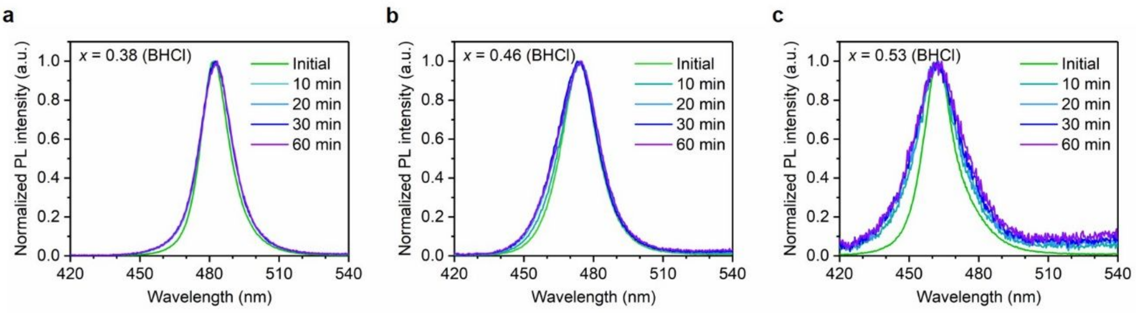

Figure S12. Normalized PL spectra of (a) $x = 0.38$ (BHCl), (b) $x = 0.46$ (BHCl), and (c) $x = 0.53$ (BHCl) perovskite films under intense and continuous laser light illumination in N_2 atmosphere. A 405 nm continuous-wave laser with a power density of ≈ 100 mW cm^{-2} is used.

Table S1 CIE coordinates and peak wavelengths of our PeLEDs at different driving voltages.

Films	CIE coordinates (*x, y*) / Peak wavelength (nm)				
	@ 4.5 V	@ 5.0 V	@ 5.5 V	@ 6.0 V	@ 6.5 V
x = 0.38	(0.061, 0.381) / 488	(0.059, 0.400) / 490	(0.057, 0.396) / 491	(0.059, 0.389) / 491	(0.059, 0.397) / 491
x = 0.38 (BHCl)	(0.065, 0.287) / 489	(0.066, 0.288) / 489	(0.068, 0.284) / 489	(0.068, 0.280) / 489	(0.067, 0.281) / 489
x = 0.46	(0.053, 0.373) / 505	(0.064, 0.375) / 503	(0.069, 0.360) / 484	(0.043, 0.321) / 481	(0.061, 0.332) / 485
x = 0.46 (BHCl)	(0.103, 0.131) / 478	(0.103, 0.135) / 478	(0.103, 0.141) / 478	(0.102, 0.149) / 478	(0.103, 0.150) / 478
x = 0.53	(0.088, 0.362) / 505	(0.093, 0.346) / 502	(0.095, 0.325) / 504	(0.093, 0.295) / 498	(0.109, 0.267) / 476
x = 0.53 (BHCl)	(0.147, 0.026) / 457	(0.147, 0.027) / 456	(0.147, 0.027) / 456	(0.144, 0.025) / 456	(0.137, 0.014) / 456
